# A roadmap for robotic-assisted sigmoid resection in diverticular disease using a Senhance™ Surgical Robotic System: results and technical aspects

**DOI:** 10.1007/s11701-019-00980-9

**Published:** 2019-06-03

**Authors:** Ibrahim Darwich, D. Stephan, M. Klöckner-Lang, M. Scheidt, R. Friedberg, F. Willeke

**Affiliations:** Department of Surgery, St. Marien Hospital Siegen, Kampenstr. 51, 57074 Siegen, Germany

**Keywords:** Robotic sigmoid resection, Senhance, Roadmap

## Abstract

Since the turn of the century, robotic-assisted colorectal surgery has been synonymous with the da Vinci^®^ robotic surgical system. We report in this study our first results in robotic-assisted sigmoid resection for diverticular disease using the Senhance™ Surgical Robotic System, while introducing a standardized roadmap for engaging the robotic arms. 12 patients underwent a sigmoid resection using the Senhance™ Surgical Robotic System. All four arms of the robotic system were engaged during all procedures according to a previously devised roadmap. A 4-trocar technique was used in all patients. Perioperative data, including those regarding technical difficulties, were collected and analyzed. Two procedures were converted into standard laparoscopy. There were no conversions to open surgery. The mean age of the patients was 62.5 years (47–79). One third of the patients were males. The mean BMI was 27 kg/m^2^ (19–38). The mean operative time, the mean console time and the mean docking time were 219 min (204–305), 149 min (124–205) and 10 min (6–15), respectively. The mean length of stay was 9 days (6–15). There was one major complication (8.3%, Clavien–Dindo IIIb). There were no mortalities. No other complications were observed. No patients were readmitted after discharge. The Senhance™ Surgical Robotic System can be used safely in sigmoid resection for diverticular disease after adequate training and systematic planning of the different steps of the procedure. Further experience is needed to judge the benefit for patient and surgeon, as well as the cost and time effectiveness.

## Introduction

Robotic-assisted colorectal surgery had been for many years synonymous to surgical procedures with the da Vinci^®^ Surgical System (Intuitive Surgical, Sunnyvale, CA, USA) [1]. Early reports confirmed the feasibility of the system; however, even after more than 15 years of application, randomized trials failed to demonstrate substantial clinical advantages [2, 3]. Clinical trials utilizing non-randomized prospective and retrospective data collection and analysis have suggested some advantages of robotic surgery in terms of protecting pelvic autonomic nerves and reducing the conversion-to-open rate compared to standard laparoscopy [4–8].

Taking the above status quo of clinical evidence in addition to the long learning curve into consideration [9], the high acquisition and running operating costs of a surgical robotic system become a financial challenge for every health care provider considering to offer this kind of surgery [10, 11].

In March 2017, our surgical department acquired the Senhance™ Surgical Robotic System (TransEnterix, Morrisville NC, USA). Reusable instruments that very much resemble those used in the standard laparoscopy were one of the reasons for selecting this system. In addition, the system provided technological features that were novel in the field of robotic surgery like haptic feedback coupled to the controllers and the manipulation capability of the robotic camera via an eye-tracking device [12, 13]. It consisted of four fully separate components or arms, each one independently controlled through the main console, allowing for more flexibility in case of an imminent conversion to standard laparoscopy. Clinical data supporting the feasibility and safety of the system were available [14–16].

Since March 2017, more than 250 procedures had been performed at our surgical department with the Senhance™ Surgical Robotic System. Clinical data describing the early experience with this system at our department have already been published [17]. Starting October 2017 through October 2018, we had performed 12 robotic-assisted sigmoid resections for diverticular disease.

In this paper, we present our data on robotic-assisted sigmoid resection while introducing a standardized roadmap for using the Senhance™ Surgical Robotic System in that regard. Simultaneously, we discuss important technical issues that have been encountered and how they have been dealt with.

## Methods

12 patients with type 2b, 3b and 3c diverticular disease [18] with the indication for elective sigmoid resection were enrolled in a prospectively collected database for robotic-assisted surgeries. An ASA score of IV (American Society of Anesthesiologists Status) was considered as an exclusion criterion, taking into account an expected prolonged operation time during the learning phase. Due to the robotic system-defined restrictions, patients with a BMI > 40 kg/m^2^ were excluded. The characteristics of the enrolled patients are represented in Table 1.

**Table 1:** Population characteristics

Variable	Value
Mean age (years)	62.5 (47–79)
Sex (F/M)	8/4
Mean BMI (kg/m^2^)	27(19–38)
ASA	II (5), III (7)

*BMI* body mass index, *ASA* American Society of Anesthesiologists Status

Patient selection for robotic surgery went according to the surgeon’s preference. Written informed consent was obtained from the patients utilizing a standardized surgical consent form (Perimed Sigmaresektion^®^). One team consisting of two surgeons performed all procedures. The operating surgeon explained the risk and possible adverse events associated with robotic-assisted surgery. Subsequently, patients had a proper time interval to decide for robotic-assisted or standard laparoscopic surgery. Informed consent for robotic-assisted surgery, pseudonymised data collection, scientific evaluation and publication was obtained from all patients.

All patients who had a robotic-assisted procedure were offered inclusion in the registry study TRUST (TransEnterix European Register Study for robotic-assisted surgical procedures in urology, abdominal surgery, thoracic surgery and gynecology). Approval had already been granted to the TRUST study (2017-463-f-S) by the ethics committee of the St. Marien-Krankenhaus Siegen (Ethics committee of the Chamber of physicians Muenster, Germany).

Each procedure was recorded for the docking, console and operative times as well as arising intraoperative technical and surgical complications. This included reasons for conversion to standard laparoscopy or to laparotomy. Additional reporting summed the number and size of trocars used, the exact points of trocar placement, the total number and kind of instruments used per arm and the angle of the endoscope used (0° or 30°). The positioning of the robot arms—designated by color—and their allocations according to the respective stage of the procedure as well as the electronic setting of the angle of compensation of the arms was also reported. Documentation included the position of the operation table, the position of the console, the quality of communication between the surgeon at the console and the assisting surgeon at the operation table and the estimated blood loss.

### Operation technique

**Fig. 1:**
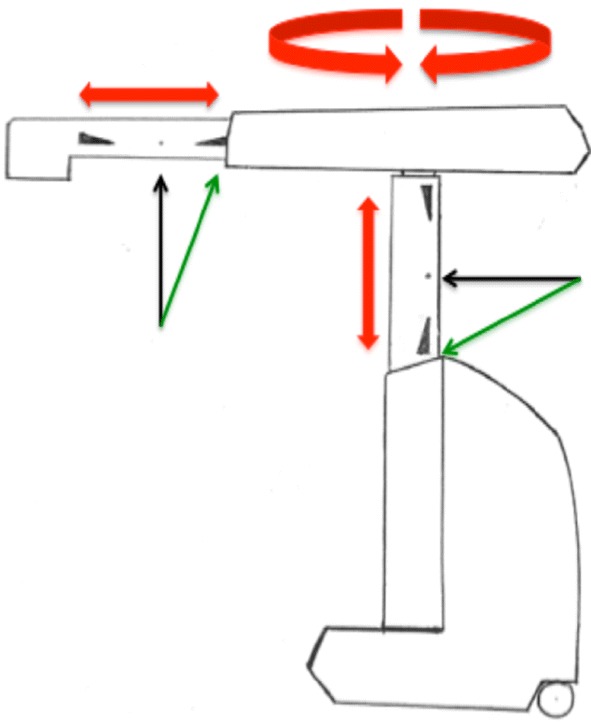
A schematic representation of the midpoints on the robotic arm extensions (black arrows). The green arrows point to the optimal starting zone of the marked midpoint. The red arrows illustrate the extent of mobility of the arm components

**Fig. 2:**
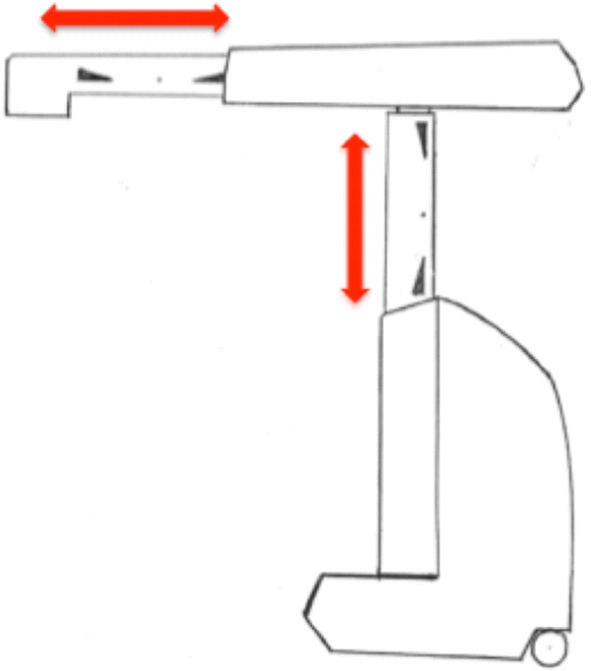
A schematic representation of the telescopic mechanism of the extending shafts. In this example the shafts are nearly fully extended

In coherence with the safety restrictions defined by TransEnterix for engaging the Senhance™ Surgical Robotic System, a standard operating procedure (SOP) was set up. According to this SOP, the patient is brought first into the standard modified lithotomy position, while securing him/her in a surgical Bean Bag Positioner under adequate padding and utilizing enough supports for the lower extremities, the buttocks and the shoulders to prevent slippage during extreme positioning, e.g., reverse Trendelenburg position. The endoscope is then driven in manually via the trocar into the abdomen. Before attaching the endoscope to the robotic arm, care is taken that the well-marked midpoints of both of the telescopic extensions are present at the rim of the vent (Fig. 1). Only this way is maximal mobility, and thus optimal visualization possible. The working instruments are then brought into the abdomen in a similar fashion under vision, while respecting the above-stated rules of engagement of the robotic arms driving those instruments. If the telescopic mechanism of the robotic arms is too extended or too retracted at the time of attachment to the instruments, limited motion is highly likely to occur (Fig. 2).

**Fig. 3:**
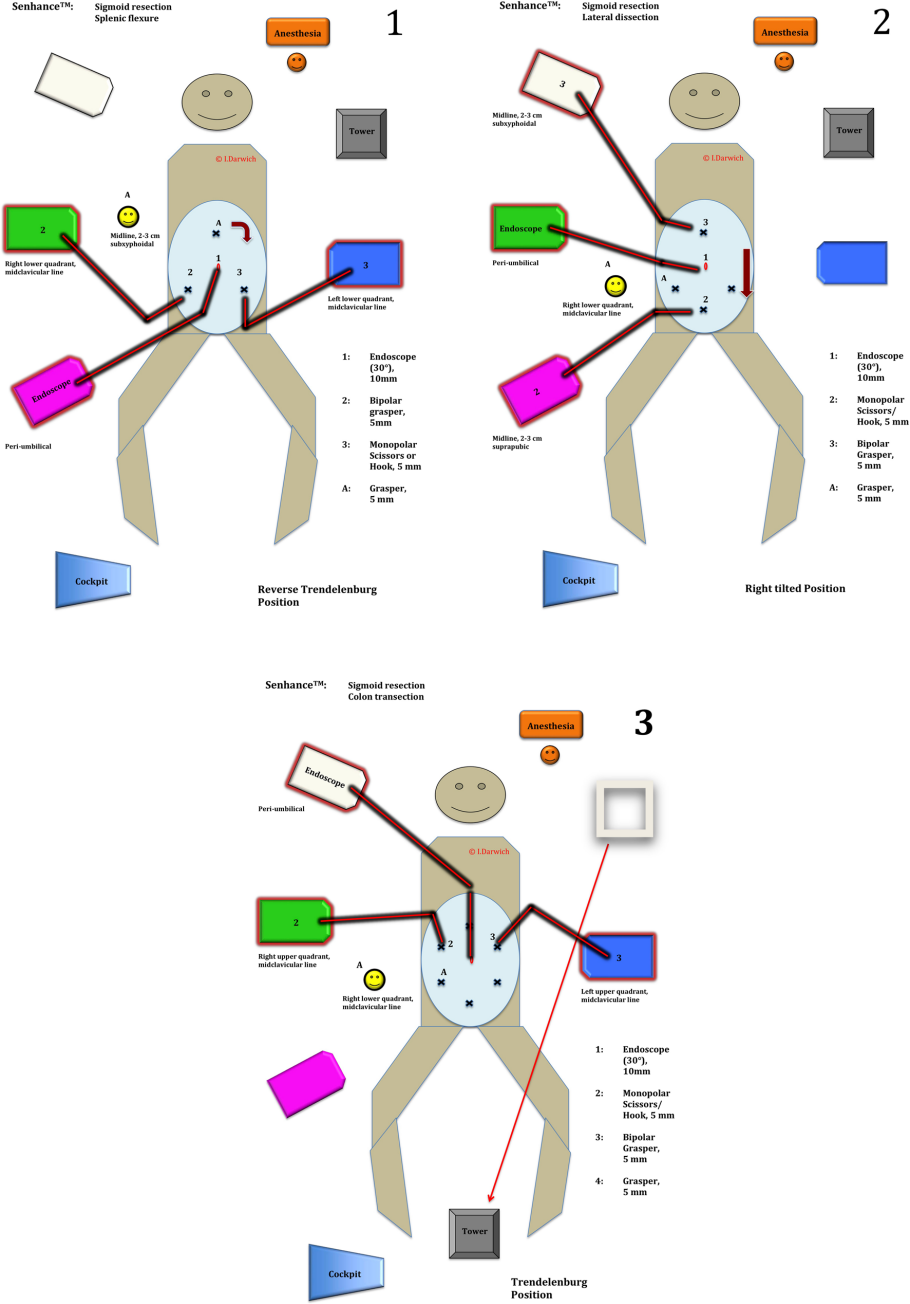
The roadmap describing the surgical sequence in robotic-assisted sigmoid resection. (1) Taking down the gastrocolic ligament and the splenic flexure. (2) Mobilizing the left colon. (3) Colon transection und tubular dissection of the sigmoid. *A* assisting surgeon

**Fig. 4:**
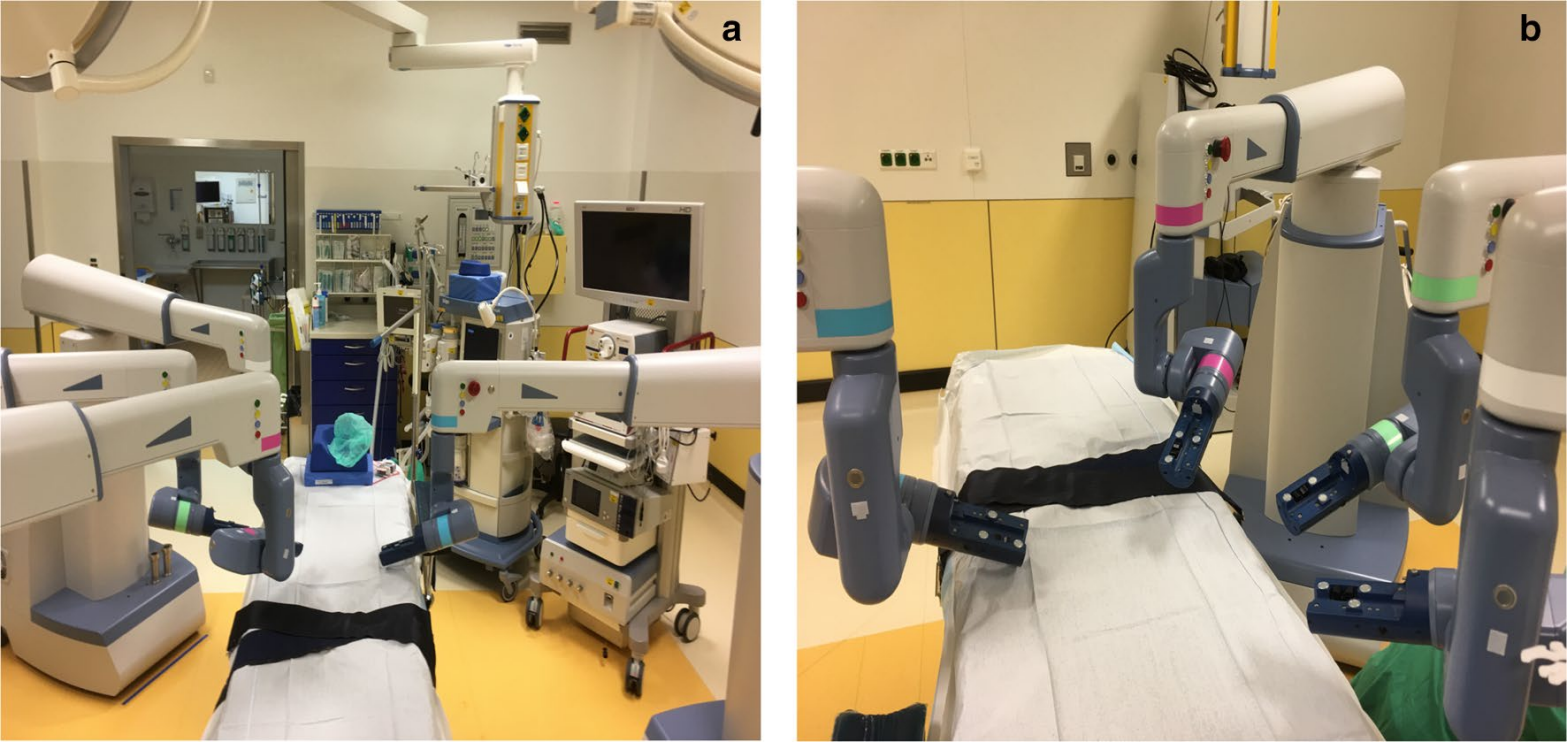
A view of the designated positions of the robot arms. **a** The arc formed array of three arms on the table’s right side with the laparoscopy tower shown on the upper left side and the fourth arm on the table’s left side; **b** a closer view of the arms with the console in the background

To standardize the procedure, we devised a roadmap defining three major steps for the operation sequence of the robotic-assisted sigmoid resection. These steps complied with our internal standard surgical technique utilized for sigmoid colectomy in diverticular disease, which involves a close to colon dissection that preserves the inferior mesenteric artery (IMA). Furthermore, this technique adheres to the recommendations of the German S2K guidelines and to evidence in the literature suggesting a lower rate of defecation disorders as well as anastomotic leakage when the IMA is preserved [18–21]. The first step of the roadmap consists of taking down the gastrocolic ligament and the splenic flexure in the reverse Trendelenburg position, the second follows with mobilizing the left colon via lateral dissection in the right tilted position and the third ends up in transecting the colon below the sacral promontory followed by close to the colon dissection of the sigmoid in the Trendelenburg position (Fig. 3). The sequence was designed in this manner so as to avoid repeated re-positioning of the robot arms (single docking). As such, three arms were designated to an arc formed array to the patient’s right side. One arm was designated to the patient’s left side (Fig. 4). Each of these steps is based on a 4-trocar technique. Three trocars are operated by the robotic arms and one operated by the assisting surgeon at the table. For each step, we predefined the exact points of placement and the sizes of the trocars, which instruments are to be used and to which robot arms they are to be allocated (Fig. 3). At the beginning of the third step, the laparoscopy tower is moved from the patient’s upper left side and brought to the lower table side to facilitate the field of vision of the assisting surgeon while engaging the left robotic arm (Fig. 3).

**Table:** Register of operated patients

Patient	Sex	Age	BMI	ASA score	LOS (days)	EBL (ml)	Docking time (min)	Console time (min)	Operative time (min)	No. of used Trocars
1	M	63	27.4	II	8	20	15	175	261	7
2	F	58	25.5	III	8	20	12	138	246	7
3	F	79	19.1	II	13	50	10	115	232	7
4	F	76	31.2	III	6	20	6	134	207	6
5	F	47	24.4	II	7	20	8	162	239	7
6	M	66	30.5	III	12	20	14	127	224	7
7	F	54	21.2	III	15	20	6	129	204	5
8	F	76	38.2	III	9	20	6	83 (S)	194	5
9	M	51	25.7	III	6	20	8	124	204	7
10	M	55	36.9	III	7	20	10	180	255	6
11	F	59	25.3	II	7	20	7	98 (C)	220	6
12	F	66	20.3	II	8	50	12	205	305	7

*BMI* body mass index, *ASA* American Society of Anesthesiologists Status, *LOS* length of stay, *EBL* estimated blood loss, *(S)* system failure, *(C)* conversion to laparoscopy

Quality of life questionnaires, including pain scores, were filled out by the patients on day 7 and day 14 after surgery and were sent back to our department. The first bowel movement was recorded. The first oral intake occurred on day of surgery in all patients in compliance with the ERAS (enhanced recovery after surgery) approach [22]. Postoperative complications were recorded and defined according to the Clavien–Dindo classification system [23].

## Results

### Patients

12 patients (8 females) with types 2b, 3b and 3c diverticular disease [18] (German S2K guidelines) of the sigmoid colon underwent a robotic-assisted sigmoid resection using the Senhance™ Surgical Robotic System. The mean age of the patients was 62.5 years (47–79). The mean BMI was 27 kg/m^2^ (19–38). 5 Patients had an ASA score II, the rest a score of III (Table 1).

### Collected perioperative data

**Fig. 5:**
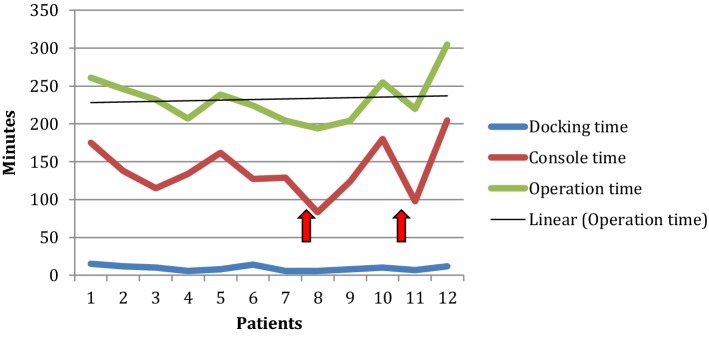
A graph summing up the docking, console und operation times. The red arrows identify conversion to standard laparoscopy

The mean docking time was 10 min (6–15). The mean console time was 149 min (124–205). The mean operative time was 219 min (204–305). A learning curve was not observed (Fig. 5). A 4-trocar technique per step was used with a mean number of six placed trocars per procedure (5–7). The mean estimated blood loss was 25 ml (20–50). The first bowel movement occurred on day 4 after surgery on average (2–7). The mean length of stay was 9 days (6–15). An overview of the results is presented in Table 2.

**Table 2:** Intraoperative and postoperative results

Variable	Mean value
Docking time (min)	10 (6–15)
Console time (min)	149 (124–205)
Operative time (min)	219 (204–305)
No. of trocars used	6 (5–7)
LOS (days)	9 (6–15)
EBL (ml)	25 (20–50)
First bowel movement (DAS)	4 (2–7)

The console und the operative times of the two procedures, which involved a robotic system failure and a conversion to laparoscopy, were omitted on purpose from the above data analysis

*LOS* length of stay, *EBL* estimated blood loss, *DAS* days after surgery

One major complication (Clavien–Dindo IIIb, 8.3%) was recorded leading to re-operation (re-laparoscopy) on the 7th postoperative day after a sudden rise of the C-reactive protein and the white blood cell count was registered (Table 3). A CT scan with rectal contrast enema revealed too much free abdominal air yet without proof of a contrast leak in the anastomotic region or elsewhere in the colon. A tiny concealed colon lesion at the splenic flexure, with no signs of thermal injury, was then identified using an intraoperative methylene blue enema and was then sutured per laparoscopy. The lesion was deemed as a diverticular rupture since the patient had a pan colonic diverticular disease. A target drain was placed and a diverting loop ileostomy was fashioned. The patient was discharged on day 15 after primary surgery. No other surgical complications were recorded. There were no mortalities.

**Table 3:** Overall morbidity

Postoperative complications	*n* (%)
Characteristic	
Colon perforation	1 (8.3)
Other	0 (0)
Classification	
Clavien–Dindo IIIb	1 (8.3)
Mortality	0 (0)

### Quality of life (QOL)

**Fig. 6:**
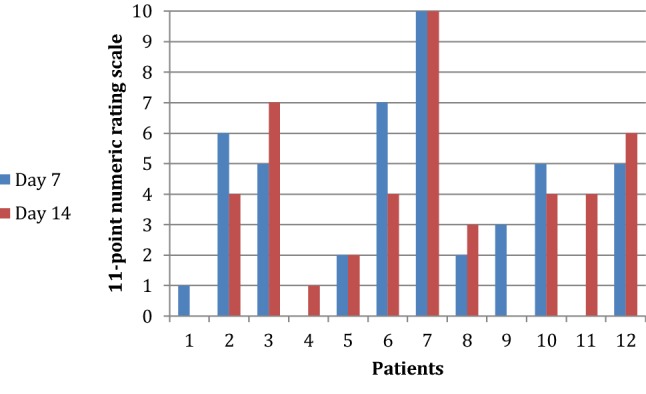
Diagram summarizing the pain scores of all patients at 7 and at 14 days after surgery

All patients filled out the QOL questionnaires. At 14 days after surgery, none of the patients felt well enough for him/her to resume normal daily life activities (job, school, household) and all stated that they needed more time to recuperate. The 11-point numeric rating scale (NRS-11) with a 100-mm visual analog scale (VAS) was utilized to characterize and quantify postoperative pain. 17% of the patients reported no pain at 7 and at 14 days after surgery. 8% of the patients reported worst pain at 7 and at 14 days after surgery. 75% of the patients reported mild to moderate pain at 7 and at 14 days after surgery. The patient’s answers are summarized in Table 4 and Fig. 6.

**Table 4:** Pain described by patients 7 days and 14 days after surgery

Extent of pain (NRS-11, VAS)	7th day after surgery No. of patients (*n*)	14th day after surgery No. of patients (*n*)
No pain (0)	2	2
(1–3)	4	3
(4–7)	5	6
(8–9)	0	0
Worst pain imaginable (7–10)	1	1

The 11-point numeric rating scale (NRS-11) with a 100-mm visual analog scale (VAS) was used in collecting the data

### Robot-specific technical difficulties

There were no conversions to laparotomy but two procedures had to be converted to standard laparoscopy. The first one was caused by a software error within the Senhance™ System. This was characterized by error messages showing “limited motion” and “exceeding force” without any recognizable problems on the hardware part. In other terms, no arm collision, overextension or maximal retraction as seen in Fig. 2 could be detected. This led us to convert to standard laparoscopy. Technical support was later able to detect a damaged arm, which had caused the system failure.

The second conversion to standard laparoscopy was due to a case of “limited motion”. The situation arose right at the beginning of the second step when the patient was brought into the right tilted position. We were unable to position the robotic arms and the dissecting instruments appropriately to perform the lateral mobilization of the colon. The dissecting instruments came in at a very steep angle into the abdomen, resulting in overextension of the vertical telescopic mechanism of the robotic arm, ultimately causing limited motion. As a result, no optimal visualization of the white line of Toldt could be obtained and dissection could not be carried out. In this particular case, the patient had a relatively short stature of 151 cm at a BMI of 25.3 kg/m^2^.

## Discussion

### Three-step roadmap

After establishing the use of the Senhance™ Surgical Robotic System in inguinal hernia repair at our department, we devised a detailed three-step roadmap for robotic-assisted laparoscopic sigmoid resection using this system. This involved the use of all four robotic arms, assigned to fixed locations in the operating theatre, while the first surgeon simultaneously engaged three arms at a time via the main console. With this approach, we started our colorectal program for robotic-assisted surgery. While such a roadmap seemed to be very helpful in increasing intraoperative workflow efficiency, this was not verifiable in terms of a clear learning curve (Fig. 5). This is probably due to the relatively low number of cases treated in this study. Another factor influencing the learning curve could have been the already acquired experience with the Senhance™ Surgical Robotic System gathered by having already performed more than 250 procedures (Hernia, upper GI tract) before proceeding to sigmoid colectomies.

We still faced technical difficulties with this surgical robotic system, which eventually resulted in two conversions to standard laparoscopy. A damaged arm caused the first conversion and that damage was not immediately evident at the start of the procedure. Future improvements in software development should be able to identify hardware malfunction in advance. The second case of conversion demonstrates that a patient with a rather short stature and a very small abdomen might not be the perfect candidate for robotic-assisted laparoscopic sigmoid resection with this robotic system. The development of wristed instruments should soon be able to improve our ability to expose and dissect within a very limited space. The ability to avoid conversion to laparotomy demonstrates the technical feasibility and safety of the procedure in general. Furthermore, conversion to laparoscopy proved to be easy since normal laparoscopic trocars are utilized with this surgical robotic system, which meant that the robotic arms were simply moved aside and standard laparoscopy was swiftly commenced.

### Complication

While the overall clinical results were encouraging, the colon perforation on day 7 at the level of the splenic flexure remains to be discussed. As in laparoscopic surgery, tissue control in robotic-assisted surgery is solely dependent on instrument manipulation. The lack of tactile feedback remains to be an issue. And while haptic feedback transmitted to the surgeon at the console is an impressive technological novelty, it obviously did not prevent such an event. Since we interpreted the perforation as a ruptured diverticulum, we would not expect to have been able to avoid this incidence with standard laparoscopic surgery [24].

### Quality of life

Excluding the patient who had a complication and rated her perceived pain with the maximum possible score at 7 as well as at 14 days after surgery, the pain scores and QOL assessment compared well to those described in literature following laparoscopic colorectal surgery [25, 26].

### Comparing Senhance™ with da Vinci^®^

The Senhance™ Surgical Robotic System brings with it novel advances in minimal invasive surgery by coupling haptic feedback to its console and introducing a camera driven by an eye-tracking system to the field of surgical robotics. The controllers resemble laparoscopic instruments while the whole setup of the system reminds much of standard laparoscopy. In addition to that, the ability to utilize conventional laparoscopic 5- and 10-mm trocars stands out in comparison to the da Vinci^®^ robotic system which incorporates its own 8- and 12-mm ports [27]. Since fully reusable instruments are used by this surgical robotic system, an advantage regarding cost efficacy is expected when compared to the da Vinci^®^ system. Excluding the acquisition costs of the Senhance™ Surgical Robotic System, the robot-specific cost per case at our department was 800 Euros, covering the expenses for drapes, instruments and maintenance according to a flat rate-based service contract. This comes in contrast to costs as high as 1700 Euros for drapes and instruments with da Vinci^®^ robotic system while excluding maintenance and acquisition costs [28].

## Conclusions

This study demonstrates the feasibility and safety of this surgical robotic system in colorectal surgery. We present in this paper a detailed demonstration of our devised surgical roadmap for performing a robotic-assisted sigmoid resection for diverticular disease using the Senhance™ robot. Yet despite this roadmap, conversion to laparoscopy occurred in 2 out of 12 patients. The introduction of novel technologies to the Senhance™ surgical robotic system like the ultrasound dissector and wristed instruments should further improve the robotic-assisted technique by increasing its dexterity.

Clearly, more data and trials are needed to further investigate this robotic system and compare it in terms of clinical outcome and cost efficacy to both standard laparoscopy and other robotic systems, namely the da Vinci^®^.

## References

[1] Weber PA, Merola S, Wasielewski A, Ballantyne GH, Delaney CP (2002). Telerobotic-assisted laparoscopic right and sigmoid colectomies for benign disease. Dis Colon Rectum.

[2] Prete FP (2018). Robotic versus laparoscopic minimally invasive surgery for rectal cancer. Ann Surg.

[3] Jayne D (2017). Effect of robotic-assisted vs conventional laparoscopic surgery on risk of conversion to open laparotomy among patients undergoing resection for rectal cancer the rolarr randomized clinical trial. J Am Med Assoc.

[4] Wang G, Wang Z, Jiang Z, Liu J, Zhao J, Li J (2017). Male urinary and sexual function after robotic pelvic autonomic nerve-preserving surgery for rectal cancer. Int J Med Robot Comput Assist Surg.

[5] Trastulli S (2012). Robotic resection compared with laparoscopic rectal resection for cancer: systematic review and meta-analysis of short-term outcome. Color Dis.

[6] Lee SH, Kim DH, Lim SW (2018). Robotic versus laparoscopic intersphincteric resection for low rectal cancer: a systematic review and meta-analysis. Int J Colorect Dis.

[7] Holmer C, Kreis ME (2018). Systematic review of robotic low anterior resection for rectal cancer. Surg Endosc Other Intervent Tech.

[8] Speicher PJ, Englum BR, Ganapathi AM, Nussbaum DP, Mantyh CR, Migaly J (2015). Robotic low anterior resection for rectal cancer. Ann Surg.

[9] Bokhari MB, Patel CB, Ramos-Valadez DI, Ragupathi M, Haas EM (2011). Learning curve for robotic-assisted laparoscopic colorectal surgery. Surg Endosc.

[10] Baek SJ, Kim SH, Cho JS, Shin JW, Kim J (2012). Robotic versus conventional laparoscopic surgery for rectal cancer: a cost analysis from a single institute in Korea. World J Surg.

[11] Steinberg PL, Merguerian PA, Bihrle W, Heaney JA, Seigne JD (2008). A da Vinci robot system can make sense for a mature laparoscopic prostatectomy program. JSLS.

[12] Rao PP (2018). Robotic surgery: new robots and finally some real competition!. World J Urol.

[13] Peters BS, Armijo PR, Krause C, Choudhury SA, Oleynikov D (2018). Review of emerging surgical robotic technology. Surg Endosc.

[14] Fanfani F (2016). Total laparoscopic (S-LPS) versus TELELAP ALF-X robotic-assisted hysterectomy: a case–control study. J Minim Invasive Gynecol.

[15] Stark M, Pomati S, D’Ambrosio A, Giraudi F, Gidaro S (2015). A new telesurgical platform—preliminary clinical results. Minim Invasive Ther Allied Technol.

[16] Spinelli A (2018). First experience in colorectal surgery with a new robotic platform with haptic feedback. Color Dis.

[17] Stephan D, Sälzer H, Willeke F (2018). First experiences with the new Senhance^®^ telerobotic system in visceral surgery. Visceral Med.

[18] Leifeld L (2014). S2 k-Leitlinie Divertikelkrankheit/Divertikulitis 1. Z Gastroenterol.

[19] Tocchi A, Mazzoni G, Fornasari V, Miccini M, Daddi G, Tagliacozzo S (2001). Preservation of the inferior mesenteric artery in colorectal resection for complicated diverticular disease. Am J Surg.

[20] Masoni L (2013). Preservation of the inferior mesenteric artery via laparoscopic sigmoid colectomy performed for diverticular disease: real benefit or technical challenge: a randomized controlled clinical trial. Surg Endosc.

[21] Borchert DH (2015). Observational study on preservation of the superior rectal artery in sigmoid resection for diverticular disease. Int J Surg.

[22] Fearon KCH (2005). Enhanced recovery after surgery: a consensus review of clinical care for patients undergoing colonic resection. Clin Nutr.

[23] Clavien PA (2009). The Clavien–Dindo classification of surgical complications: five-year experience. Ann Surg.

[24] Kirchhoff P, Clavien PA, Hahnloser D (2010). Complications in colorectal surgery: risk factors and preventive strategies. Patient Saf Surg.

[25] Jayne DG (2007). Randomized trial of laparoscopic-assisted resection of colorectal carcinoma: 3-year results of the UK MRC CLASICC trial group. J Clin Oncol.

[26] Siassi M, Weiss M, Hohenberger W, Lösel F, Matzel K (2009). Personality rather than clinical variables determines quality of life after major colorectal surgery. Dis Colon Rectum.

[27] Bae SU, Baek SJ, Hur H, Baik SH, Kim NK, Min BS (2015). Robotic left colon cancer resection: a dual docking technique that maximizes splenic flexure mobilization. Surg Endosc.

[28] van Dam P-J (2011). Are costs of robot-assisted surgery warranted for gynecological procedures?. Obstet Gynecol Int.

